# Preparation of Magnetic g-C_3_N_4_/Fe_3_O_4_ Composite and Its Application in the Separation of Catechol from Water

**DOI:** 10.3390/ma12182844

**Published:** 2019-09-04

**Authors:** Ruilan Xu, Yong Peng

**Affiliations:** School of Science, Nanchang Institute of Technology, Nanchang 330099, China

**Keywords:** catechol, g-C_3_N_4_, chemical co-precipitation, magnetic separation, adsorption

## Abstract

Catechol has strong toxicity and deformity as well as carcinogenicity, and it is difficult to degrade naturally. Therefore, it is of great practical significance to develop efficient adsorbents to separate catechol from water quickly and effectively. In this work, g-C_3_N_4_/Fe_3_O_4_ magnetic nanocomposites were prepared using g-C_3_N_4_ as the matrix by chemical co-precipitation, mixing with Fe^2+^ and Fe^3+^ solutions. Then, g-C_3_N_4_/Fe_3_O_4_ was used, for the first time, as an adsorbent to investigate the removal rate of catechol under different conditions by the magnetic field separation method. The adsorption parameters of the g-C_3_N_4_/Fe_3_O_4_ nanocomposite were evaluated by the Langmuir and Freundlich adsorption models. The results showed that the g-C_3_N_4_/Fe_3_O_4_ nanocomposite presented a two-step adsorption behavior and a considerably high adsorption capacity. The removal rate of catechol reached 70% at the dosage of 50 mg, adsorption time of 30 min, and pH value of 6. Five adsorption–desorption cycles demonstrated that the g-C_3_N_4_/Fe_3_O_4_ material had good stability and reusability.

## 1. Introduction

Catechol (1,2-dihydroxybenzene) is an important chemical product, which is used widely as the intermediate of fine chemicals such as pesticides, pharmaceuticals, and dyes. Moreover, catechol is also an important intermediate product of phenolic degradation. These are the main reasons why catechol has become an important component of pollutants in environmental water [1]. Due to its strong toxicity, deformity, and carcinogenicity, catechol has been included in the list of priority pollutants in many countries [2,3,4]. Furthermore, catechol is difficult to degrade, so conventional water treatment technology cannot effectively remove it from polluted water [5]. Currently, the common methods for the removal of catechol include catalytic degradation [6,7], advanced oxidation technology [8,9,10,11], and adsorption [12]. Thereinto, adsorption has become a widely used method for the treatment of catechol due to its simple operation, low cost, and high efficiency. Now, many materials are used as adsorbents to remove catechol from water such as activated carbon [12,13], dolomite [14], montmorillonite [15], hydroxyapatite [16], organophilic-bentonite [17], hematite [18], goethite [19], rutile TiO_2_ [20], α-alumina [21], magnetic vermiculite [22], resin [23], and waste Fe(III)/Cr(III) hydroxide [24]. These aforementioned materials were modified to increase the pore structure, specific surface area, or special functional groups, thereby enhancing their adsorption effect. Although these modified adsorbents presented good adsorption effect, they were still found to have some disadvantages, for example, high-cost materials, long adsorption time, difficult separation, regeneration, and so on. In order to remove catechol from water easily and effectively, a good adsorbent is still needed [14,15]. Therefore, it is of great significance to develop a new adsorbent with special properties for the rapid separation of catechol in water.

In recent years, the research and development of non-metallic materials such as carbon materials represented by graphene and graphene oxides as well as carbon and nitrogen materials represented by carbon nitride and nitrogen-doped graphene have received great attention. Thereinto, g-C_3_N_4_ (i.e., graphite-phase C_3_N_4_) is of particular interest. Its unique two-dimensional Graph-like layered stacking structure and sp^2^ hybrid π conjugated electron band structure can form strong interactions with analytes such as hydrogen bonds, π–π stacking, electrostatic interactions, Van der Waals forces, and hydrophobic effects, which guarantee g-C_3_N_4_ as an excellent adsorbent [25,26,27]. However, g-C_3_N_4_ cannot be used directly as an adsorbent because it is prone to reagglomeration when separated from homogeneous solutions. In addition, the good dispersion of g-C_3_N_4_ nanomaterials makes separation and recycling difficult [28]. g-C_3_N_4_/Fe_3_O_4_ magnetic nanocomposite prepared by grafting Fe_3_O_4_ onto C_3_N_4_ has good stability, large surface area, and good water solubility. It can also hopefully be used as a solid phase extraction adsorbent, separating the target objects from solutions quickly by an external magnetic field. This will greatly simplify cumbersome procedures such as centrifugation and filtration, thus exhibiting significant practical significance. The reported methods of the preparation of g-C_3_N_4_/Fe_3_O_4_ composite contain chemical co-precipitation [28,29,30], the solvothermal method [27,31], and simple physical blending [32]. Wang et al. [28] successfully prepared g-C_3_N_4_/Fe_3_O_4_ nanocomposites by chemical co-precipitation, which was used as a solid phase extraction adsorbent for the first time to separate and enrich polycyclic aromatic hydrocarbons (PAHs) in environmental water. The detection limit of this method was 0.05–0.1 µg/L, the precision was 1.8–5.3%, and the recovery rate was 80.0–99.8%. Zheng et al. [32] developed a solid-phase microextraction method based on magnetic g-C_3_N_4_ nanosheets, which were further applied for the determination of PAHs in different edible oil samples coupled with GC/MS analysis. Limits of quantitation for the eight PAHs ranged from 0.4 to 0.9 ng/g. The recoveries ranged from 91.0% to 124.1%, with RSDs of less than 10.2%.

In this work, g-C_3_N_4_ was prepared by thermal polymerization with melamine as the precursor. Then, g-C_3_N_4_/Fe_3_O_4_ magnetic nanocomposites were prepared by the chemical co-precipitation of g-C_3_N_4_ with FeCl_3_·6H_2_O and FeSO_4_·7H_2_O solutions in a certain proportion. The removal rates of catechol by g-C_3_N_4_/Fe_3_O_4_ magnetic composite were investigated under different g-C_3_N_4_/Fe_3_O_4_ dosage, adsorption time, and pH value. The stability and reusability of the composites were evaluated by adsorption–desorption cycle experiments.

## 2. Materials and Methods 

### 2.1. Materials

Melamine (≥99.0%), FeCl_3_·6H_2_O (≥99.0%), FeSO_4_·7H_2_O (99.0~101.0%), concentrated ammonia water (25.0~28.0%), anhydrous ethanol (≥99.7%), isopropanol (≥99.7%), and acetic acid (≥99.8%) were purchased from Sinopharm Chemical Reagent Co. Ltd. (Shanghai, China). Acetonitrile (99.9%), methanol (99.9%), and catechol (99%) were obtained from J&K Co. Ltd. (Beijing, China). All other solvents were of HPLC or analytical grade. Deionized water was used in all experiments.

### 2.2. Preparation of Catechol Stock Solution

A concentration of 1 g/L stock solution of catechol was prepared by dissolving catechol (99%) in deionized water, then transferred into a brown bottle, and reserved in a refrigerator for later use. Working solutions were prepared daily by diluting the stock solution with deionized water.

### 2.3. Preparations of g-C_3_N_4_, Fe_3_O_4_ and g-C_3_N_4_/Fe_3_O_4_

g-C_3_N_4_ was prepared at 520 °C for 4 h in a N_2_ atmosphere by thermal polymerization using melamine as the precursor, with the heating rate of 4 °C/min. After cooling, a pale yellow powder g-C_3_N_4_ was obtained by grinding. g-C_3_N_4_/Fe_3_O_4_ was prepared by chemical co-precipitation [28,29,30]. According to a typical procedure, g-C_3_N_4_ (1.860 g) was dispersed in 40.0 mL brown-yellow solution mixed of FeCl_3_·6H_2_O (0.540 g) and FeSO_4_·7H_2_O (0.278 g) and then ultrasonically dispersed for 30 min at room temperature. The resulting orange-yellow suspension was transferred to a three-necked flask in a N_2_ atmosphere at 70 °C with magnetic stirring for 1 h. Next, the ammonia solution was injected into the reaction mixture until the pH reached 10.0. The resulting mixture was stirred at 70 °C for another 1 h, after which the reaction mixture was cooled and washed several times with ethanol and deionized water. The nanocomposite was dried in a vacuum oven at 40 °C for 12 h. 

In order to better characterize and prove the structure of g-C_3_N_4_/Fe_3_O_4_, the Fe_3_O_4_ nanoparticles were prepared by chemical co-precipitation similar to the preparation of g-C_3_N_4_/Fe_3_O_4_. FeCl_3_·6H_2_O (4.066 g) and FeSO_4_·7H_2_O (2.107 g) were dissolved in 80 mL water ultrasonically at room temperature and then transferred into a three-necked flask in a N_2_ atmosphere at 70 °C with magnetic stirring for 1 h. Next, the ammonia solution was injected into the reaction mixture until the pH reached 10.0. The resulting mixture was stirred at 70 °C for another 1 h, after which the reaction mixture was cooled and washed several times with deionized water. The nanoparticle was dried in a vacuum oven at 40 °C for 12 h. 

### 2.4. Characterizations of g-C_3_N_4_, Fe_3_O_4_ and g-C_3_N_4_/Fe_3_O_4_

X-ray diffraction (XRD) was carried out on Bruker D8 Advance Diffraction Instrument (Bruker, Karlsruhe, Germany) in the range of 10°–80° at a scanning rate of 5°/min under 40 kV. Scanning electron microscopy (SEM) and transmission electron microscopy (TEM) were executed on a JEOL JSM-6701F (JEOL, Tokyo, Japan) and JEM-2100 (Hitachi, Tokyo, Japan), respectively. The Fourier transform infrared (FT-IR) spectrum was obtained from a Thermo Fisher Nicolet iS10 (Thermo Fisher Scientific, Waltham, MA, USA) in the range of 400–4000 cm^−1^ with KBr pellets. Nitrogen adsorption−desorption isotherms were measured on a Micromeritics ASAP 2020 (Micromeritics, Norcross, GA, USA). Magnetic characterization was carried out using a Physical Property Measurement System (PPMS-9, Quantum design, San Diego, CA, USA) with fields up to 20,000 Oe at the temperature of 300 K.

### 2.5. Adsorption Properties of g-C_3_N_4_/Fe_3_O_4_

**Figure 1:**
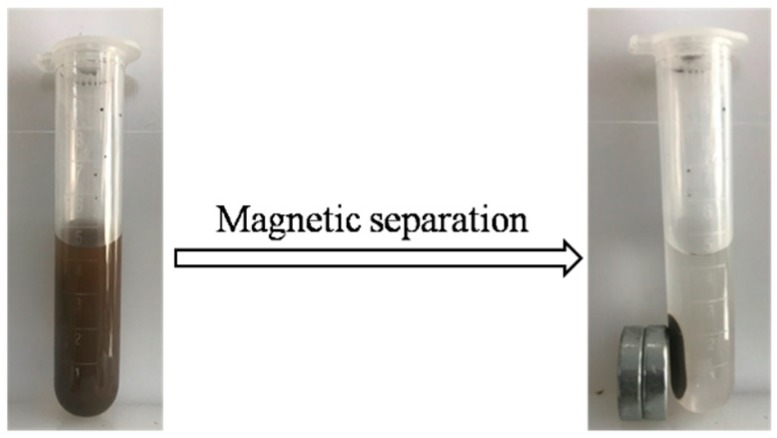
Separation of the supernatant by an external magnetic field.

Adsorption isotherms of catechol onto g-C_3_N_4_/Fe_3_O_4_ surfaces were carried out in a capped centrifugal tube at 308 K with pH 6 by ultrasonic adsorption. A series of catechol solutions with concentrations from 6 mg/L to 300 mg/L were prepared from a stock solution of 1 g/L. The pH of the solutions was adjusted with acetic acid and ammonia solution. A total of 50 mg of the g-C_3_N_4_/Fe_3_O_4_ composites were added into 5 mL catechol standard solutions with initial concentrations as above, respectively, followed by ultrasonic adsorption for 30 min in room temperature. An external magnetic field was used to separate the g-C_3_N_4_/Fe_3_O_4_ nanocomposites from the catechol supernatant (as shown in Figure 1). The concentration of catechol solution was determined by an Ultraviolet-Visible spectrophotometer (UV-1810S, Yoke, Shanghai, China) with the maximum absorption wavelength of 275 nm.

The adsorption properties of the g-C_3_N_4_/Fe_3_O_4_ nanocomposites on catechol were investigated under different g-C_3_N_4_/Fe_3_O_4_ dosage, adsorption time, and pH values (as shown in Table 1). The removal rate (*η*) and adsorption capacity (*Q_e_*) was calculated according to the difference between the initial concentration of catechol and the concentration of catechol in the supernatant at adsorption equilibrium. The calculation formula is as follows:

*η* = (*C*_0_ − *C_e_*)/*C*_0_ × 100% (1)

*Q_e_* = (*C*_0_ − *C_e_*)*V*/*m* (2)

where *C*_0_ and *C_e_* (mg/L) are the initial concentration of catechol in water and the concentration of catechol in the supernatant at adsorption equilibrium, respectively. *m* (mg) is the dosage of g-C_3_N_4_/Fe_3_O_4_ and *V* (mL) is the volume of the aqueous solution.

Acetonitrile, methanol, and isopropanol were chosen as desorption agents to study the effects on the recovery of catechol from g-C_3_N_4_/Fe_3_O_4_ nanocomposites under the optimum adsorption conditions. A total of 5 mL of the three desorption solvents above-mentioned were added with ultrasonic desorption for 20 min. Five adsorption and desorption experiments were repeated to test the stability and reusability of the g-C_3_N_4_/Fe_3_O_4_ nanocomposite. The concentration of catechol in the supernatant after desorption was determined to be *C_d_* (mg/L), and the formula for calculating the recovery rate of catechol was as follows:

*δ* = *C_d_*/(10 − *C_e_*) × 100% (3)

### 2.6. Adsorption Isotherms

The adsorption isotherms were subjected to analysis in terms of the well-known Langmuir and Freundlich adsorption models. 

The Langmuir equation can be represented by [33]:

*C*_e_/*Q*_e_ = 1/(*Q*_m_·*K*_L_) + *C*_e_/*Q*_m_ (4)

where *C*_e_ (mg/L) is the concentration of catechol at equilibrium; *Q*_e_ (mg/g) is the equilibrium amount of catechol adsorbed; *K*_L_ (L/mg) is a constant related to the intensity of adsorption; and *Q*_m_ (mg/g) is the maximum amount adsorbed for a complete monolayer coverage.

The equation of the Freundlich isotherm can be written as [34]:

log*Q*_e_ = log*K*_F_ + (1/*n*)·log*C*_e_ (5)

where *K*_F_ (L/mg) and *n* are the Freundlich constants related to the adsorption capacity and the intensity of adsorption, respectively.

## 3. Results and Discussion

### 3.1. Characterizations

**Figure 2:**
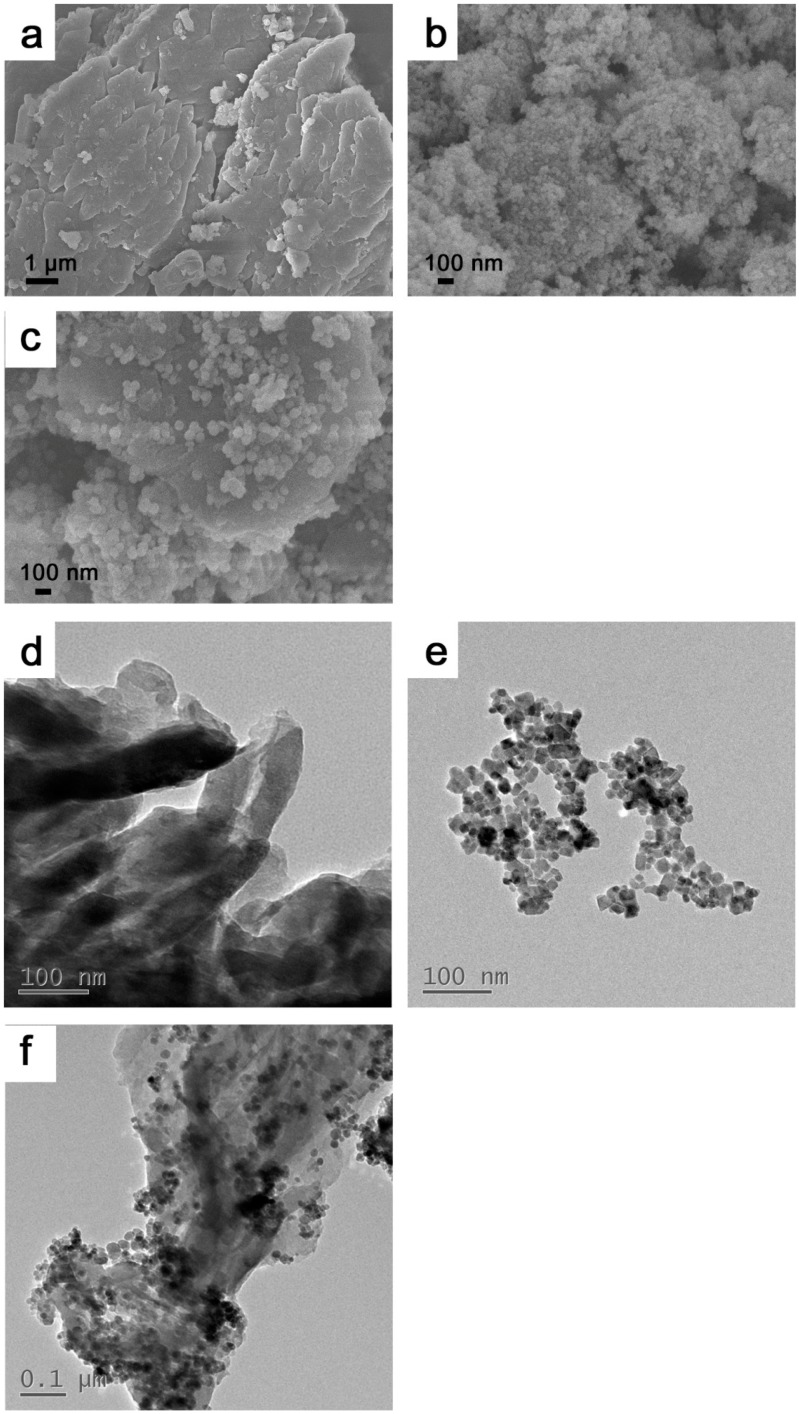
Scanning electron microscopy (SEM) images of g-C_3_N_4_ (**a**), Fe_3_O_4_ (**b**), and g-C_3_N_4_/Fe_3_O_4_ (**c**), and transmission electron microscopy (TEM) images of g-C_3_N_4_ (**d**), Fe_3_O_4_ (**e**), and g-C_3_N_4_/Fe_3_O_4_ (**f**).

Figure 2 shows the SEM images of g-C_3_N_4_ (a), Fe_3_O_4_ (b), g-C_3_N_4_/Fe_3_O_4_ (c), and TEM images of g-C_3_N_4_ (d), Fe_3_O_4_ (e), and g-C_3_N_4_/Fe_3_O_4_ (f). As shown in Figure 2a,d, g-C_3_N_4_ prepared by melamine had a lamellar structure with a relatively smooth surface. Figure 2b,e shows that most of the Fe_3_O_4_ nanoparticles were spherical and uniform in size, with an average particle size of about 20 nm, but poorly dispersed. Figure 2c,f shows that Fe_3_O_4_ particles were successfully loaded onto the lamellar structure of g-C_3_N_4_, forming a g-C_3_N_4_/Fe_3_O_4_ composite system.

**Figure 3:**
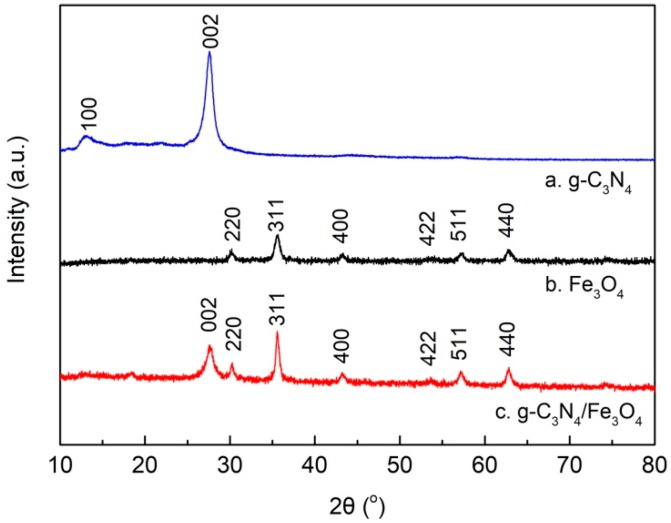
X-ray diffraction (XRD) patterns of g-C_3_N_4_ (**a**), Fe_3_O_4_ (**b**), and g-C_3_N_4_/Fe_3_O_4_ (**c**).

Figure 3 shows the XRD patterns of g-C_3_N_4_ (a), Fe_3_O_4_ (b), and g-C_3_N_4_/Fe_3_O_4_ (c). According to the literature [28,30], the XRD spectra of pure g-C_3_N_4_ has two characteristic peaks at 2*θ* = 27.4° and 2*θ* = 13.1°. Therefore, the strong peak at 27.4° can be attributed to the typical (002) interlayer diffraction peak, while the peak at 13.1° belongs to the (100) crystal plane diffraction peak accumulated in the interlayer structure. As shown in Figure 3a, the characteristic peaks of g-C_3_N_4_ prepared by melamine were consistent with the data stated above and the crystal database JCPDS 87-1526, with no other impurity peaks, indicating that g-C_3_N_4_ was successfully prepared. In Figure 3b, six characteristic diffraction peaks of (220), (311), (400), (440), (422), (511), and (440) could be observed at the 2*θ* degree of 30.2°, 35.6°, 43.3°, 53.5°, 57.1°, and 62.7°, respectively, which are in accordance with the standard Fe_3_O_4_ crystal data (JCPDS 19-0629) and literature description [28,30]. The diffraction peaks identified that the Fe_3_O_4_ had a face-centered-cubic structure [29]. In Figure 3c, both of the aforementioned characteristic diffraction peaks of g-C_3_N_4_ and Fe_3_O_4_ appeared in one XRD pattern at the same time, indicating that material c was a two-phase composite composed of g-C_3_N_4_ and Fe_3_O_4_. Furthermore, the intensity of the Fe_3_O_4_ diffraction peaks in the composite were higher than that in the pure Fe_3_O_4_ powder. Thus, the dispersion degree of Fe_3_O_4_ nanoparticles in the composite was significantly improved, which is beneficial for an increase in the adsorption sites and therefore the adsorption capacity.

**Figure 4:**
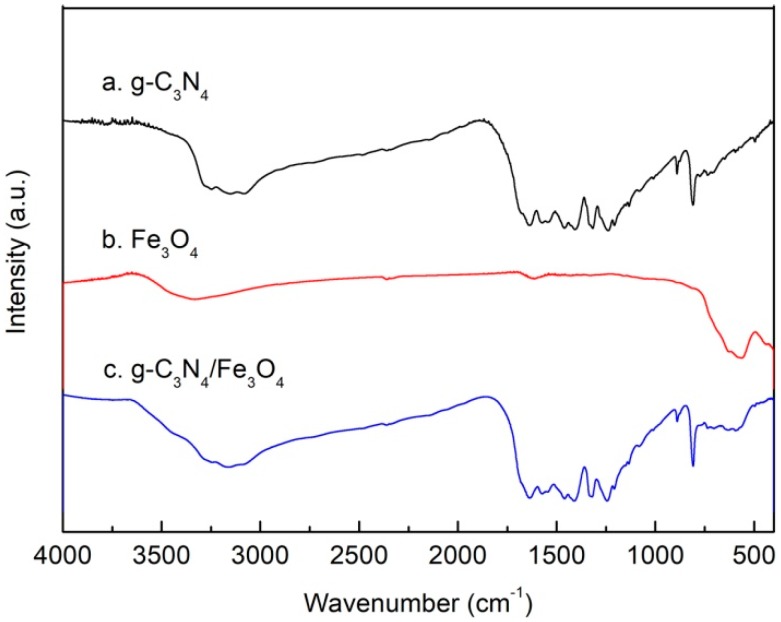
IR spectrum of g-C_3_N_4_ (**a**), Fe_3_O_4_ (**b**), and g-C_3_N_4_/Fe_3_O_4_ (**c**).

Figure 4 shows the IR spectrum of g-C_3_N_4_ (a), Fe_3_O_4_ (b), and g-C_3_N_4_/Fe_3_O_4_ (c). In Figure 4a, the broad absorption band at 3100–3300 cm^−1^ can be attributed to the stretching vibration of N–H in g-C_3_N_4_. Absorption peaks at 1640–1400 cm^−1^ can be assigned to the stretching vibration of repetitive elements in g-C_3_N_4_. Absorption peaks at 1332 cm^−1^ and 1244 cm^−1^ are due to the stretching vibration of C–N, and the absorption peaks at 808 cm^−1^ can be attributed to the bending vibration of triazine ring. In Figure 4b, the characteristic absorption peak around 570 cm^−1^ belongs to the stretching vibration of the Fe–O bond in pure Fe_3_O_4_, which is in accordance with the data in the literature [28,30]. The IR spectrum of Figure 4c shows that there are absorption peaks corresponding to Figure 4a at 3100–3300 cm^−1^, 1640–1400 cm^−1^, 1333 cm^−1^, 1246 cm^−1^, and 808 cm^−1^, indicating that material b is composed of g-C_3_N_4_. In addition, the peak at around 570 cm^−1^ was also consistent with the characteristic absorption peak of Fe–O in Fe_3_O_4_, which further proved that the g-C_3_N_4_/Fe_3_O_4_ composite was successfully prepared.

**Figure 5:**
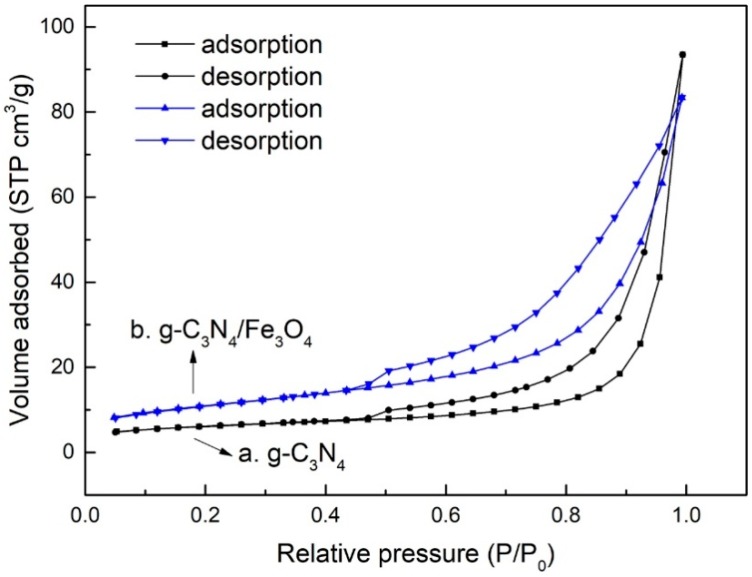
Nitrogen adsorption–desorption isotherm plots for g-C_3_N_4_ (**a**) and g-C_3_N_4_/Fe_3_O_4_ (**b**).

Nitrogen adsorption–desorption measurements (Figure 5) were performed to investigate the Brunauer-Emmett-Teller (BET) surface area (S_BET_), pore volume and dimension of pure g-C_3_N_4_ and g-C_3_N_4_/Fe_3_O_4_ composite. These textural properties are listed in Table 2. As seen from Table 2, the calculated S_BET_ of g-C_3_N_4_ showed (20.6 m^2^/g), a considerably high surface area when compared with other reported S_BET_ (8.56 m^2^/g) of g-C_3_N_4_ [29]. g-C_3_N_4_/Fe_3_O_4_ exhibited a S_BET_ of 38.8 m^2^ g^−1^, which is higher than that of the pure g-C_3_N_4_. The pore volume and size were decreased after the introduction of Fe_3_O_4_, illustrating that the Fe_3_O_4_ nanoparticles entered into the mesopores inside the g-C_3_N_4_.

**Figure 6:**
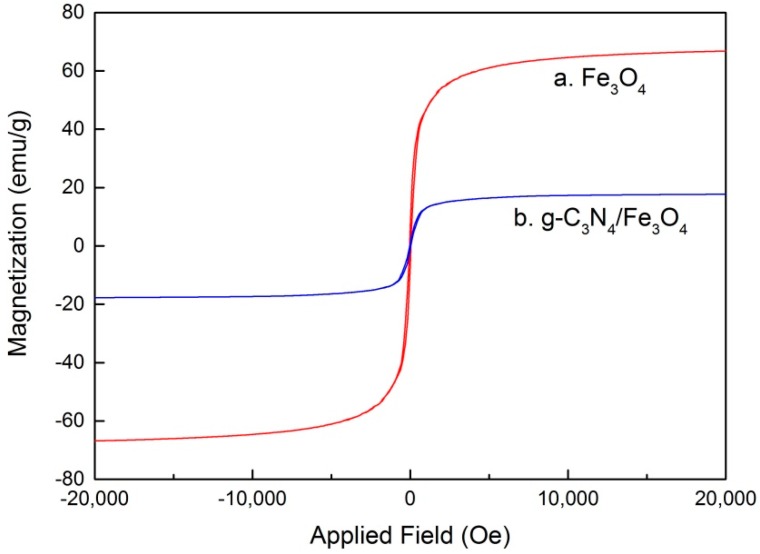
Magnetization curves of Fe_3_O_4_ (**a**) and g-C_3_N_4_/Fe_3_O_4_ (**b**).

The typical magnetization curves of Fe_3_O_4_ (a) and g-C_3_N_4_/Fe_3_O_4_ (b) at 300 K are shown in Figure 6. It can be seen that the saturation magnetization (Ms) values for Fe_3_O_4_ and g-C_3_N_4_/Fe_3_O_4_ were about 66.8 and 17.7 emu/g, respectively, indicating that the g-C_3_N_4_/Fe_3_O_4_ composite presented good magnetic property. Therefore, the g-C_3_N_4_/Fe_3_O_4_ particles could be simply and quickly separated from the aqueous solutions by an external magnetic field. 

### 3.2. Adsorption of Catechol

**Figure 7:**
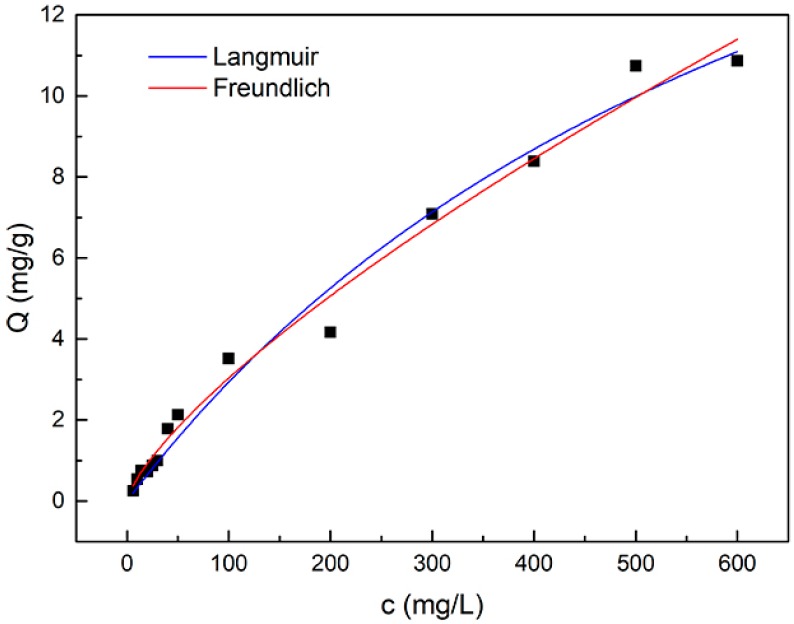
Adsorption isotherms of catechol on the g-C_3_N_4_/Fe_3_O_4_ composite. Lines are the model correlations.

The adsorption isotherm of catechol on g-C_3_N_4_/Fe_3_O_4_ composite was determined by varying the initial catechol concentration at a pH value of 6 and temperature of 308 K. As shown in Figure 7, the isotherm does not exhibit a typical type-I adsorption isotherm, according to the Brunauer classification, but a two-step adsorption behavior can be seen. This may contribute to the two different kinds of adsorption site that exist in the g-C_3_N_4_/Fe_3_O_4_ composite, since there are two potential adsorptive phases: the graphite phase and magnetite. Langmuir and Freundlich models were utilized to fit the experimental isotherm data (Figure 7). The extracted values of the adsorption isotherm parameters are summarized in Table 3. The R^2^ extracted from both models was higher than 0.98. This means that the correlated isotherms from both models were fairly good. The maximum adsorption amount (*Q*_max_) calculated from the Langmuir model was 24.9 mg g^−1^. The slightly higher R^2^ value extracted from the Freundlich model indicates that this model may describe the adsorption behavior of catechol on the g-C_3_N_4_/Fe_3_O_4_ composite better. The relatively high value of 1/*n* (0.739) implies that the two possible kinds of adsorption sites are mainly homogeneous.

**Figure 8:**
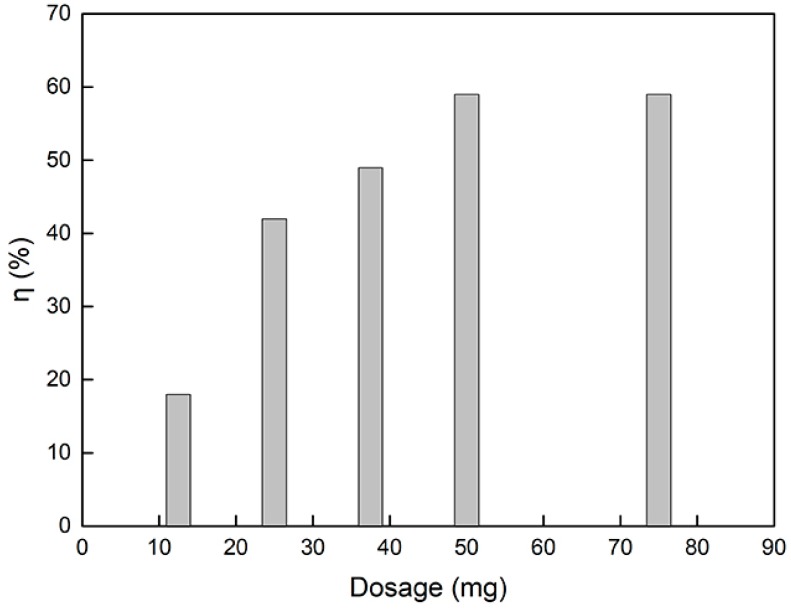
Effect of g-C_3_N_4_/Fe_3_O_4_ dosage on the catechol removal rate.

Figure 8 shows the relationship between the removal rate of catechol and g-C_3_N_4_/Fe_3_O_4_ dosage. The removal rate of catechol increased with the increase in material dosage. When the dosage was 50 mg, the removal rate reached 59%. Then, by continuing to increase the amount of g-C_3_N_4_/Fe_3_O_4_, the removal rate of catechol remained basically at the same value, which can be attributed to the adsorption equilibrium between g-C_3_N_4_/Fe_3_O_4_ and catechol.

**Figure 9:**
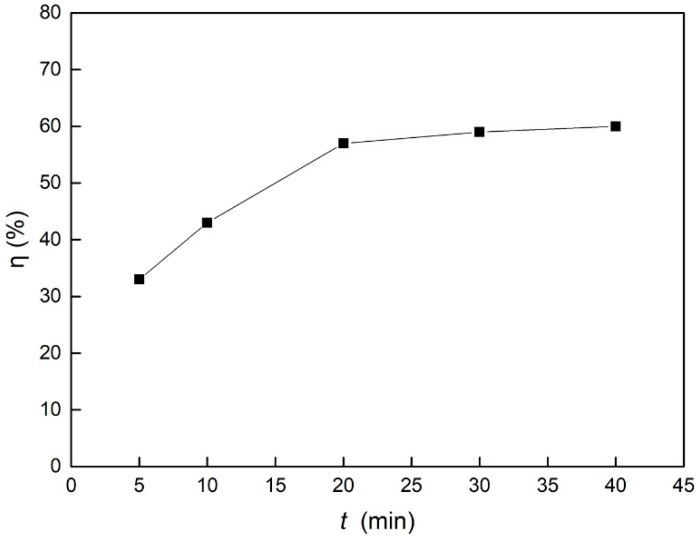
Effect of adsorption time on the catechol removal rate.

The relationship between the adsorption time and the removal rate of catechol is shown in Figure 9. It can be seen from the figure that the removal rate of catechol increased continuously with the progress of ultrasonic adsorption. When the adsorption time was 30 min, the removal rate reached 59%. When the ultrasonic adsorption was continued, the adsorption tended to be stable, and the removal rate basically remained at the same value.

**Figure 10:**
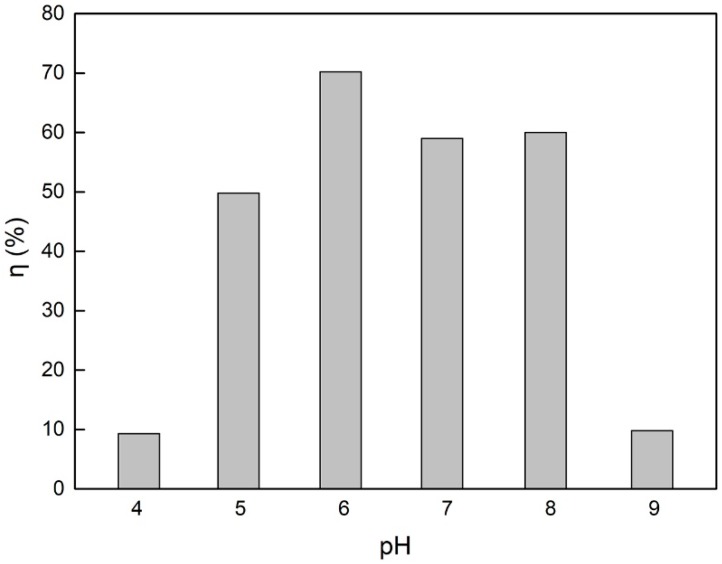
Effect of pH on the catechol removal rate.

The removal rates of g-C_3_N_4_/Fe_3_O_4_ for catechol under different pH were determined and presented in Figure 10, where the removal rate of catechol increased gradually with the increase in the solution pH. At a pH value of 6, the removal rate of catechol reached 70%. Then, with the increase in pH, the removal rate decreased slightly at 7 and 8, while at a pH value of 9, the removal rate decreased significantly. This suggests that the adsorption of catechol by g-C_3_N_4_/Fe_3_O_4_ materials may be the result of the interaction between charged groups (as described in Table 4 [26,35,36]). The amine groups on the surface of the g-C_3_N_4_/Fe_3_O_4_ act as proton acceptors because of the free lone pair electrons on the nitrogen atom and acquire positive surface charges [36]. At the same time, primary and secondary amine groups react with hydroxyl ions, making the g-C_3_N_4_/Fe_3_O_4_ particles negatively charged [26,35]. Under weak acid, weak base, and neutral conditions, catechol mainly presents in its molecular form, so hydrogen bonds are formed between the phenolic hydroxyl groups in catechol and the amino groups in g-C_3_N_4_/Fe_3_O_4_ materials, leading to its strong adsorption capacity. However, under strong alkaline conditions, the surfaces of the materials are negatively charged due to their deprotonation, while most of the catechol molecules are ionized, leading to the reduction in the adsorption capacity amount. This could be caused by the electrostatic repulsion between the deprotonated material and ionized catechol, thus affecting the recovery rate of the magnetic solid phase extraction. Suresh et al. [12] reported that the interaction of catechol with granular activated carbon at neutral pH occurred through hydrogen bonding. Moreno-Piraján et al. [13] and Khalfa [14] found that the best adsorption of catechol on activated carbon and dolomite both occurred at pH 7. These reported results of pH are similar to the results in this experiment. Therefore, the optimal pH value of the solution is 6. 

### 3.3. Desorption of Catechol

**Figure 11:**
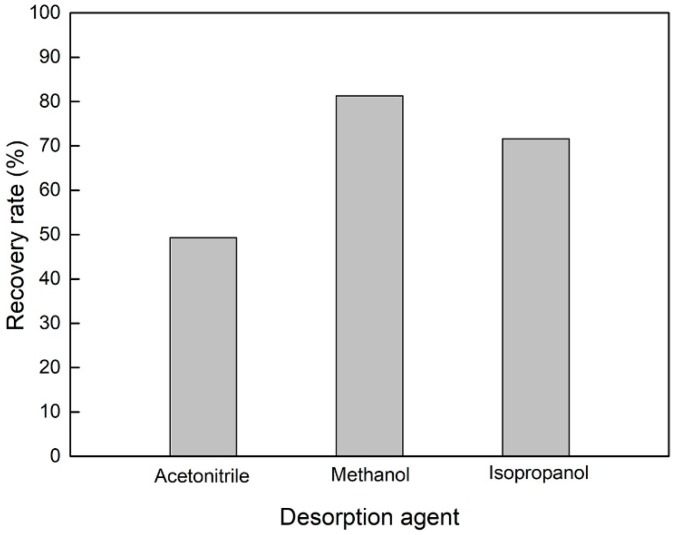
Effect of desorption agent on the recovery of catechol.

Acetonitrile, methanol, and isopropanol were added as desorption agents to investigate the desorption capacities for catechol. As can be seen from Figure 11, the desorption rates of the three desorbents were not very low, most likely due to their similar high polarity to catechol and the presence of the lone pairs of electrons, which is beneficial to the affinity of catechol. Among the three desorption agents, methanol had the highest recovery rate of 82%, followed by isopropanol with a recovery rate of 72%, and acetonitrile had the lowest recovery rate of 49%. This might be because methanol and isopropanol possess hydroxyl groups that can form hydrogen bonds with catechol, which is more easily eluted than acetonitrile. Therefore, methanol was selected as the desorption agent in this work.

**Figure 12:**
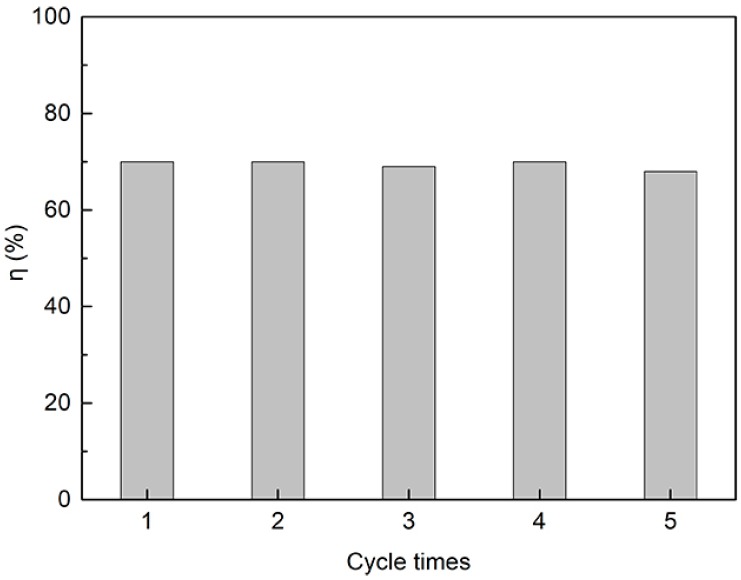
The reusability of the g-C_3_N_4_/Fe_3_O_4_ composite.

To investigate the recyclability of g-C_3_N_4_/Fe_3_O_4_, five adsorption and desorption experiments were developed under the optimum conditions obtained earlier. As shown in Figure 12, after five cycles, the removal rate of catechol by the g-C_3_N_4_/Fe_3_O_4_ composite remained at about 70%, which indicates that the material has good stability and reusability.

### 3.4. Comparison with Other Adsorbents

The maximum adsorption capacity (*Q*_m_), contact time (*t*_e_), and the BET surface area (*S*_BET_) of different adsorbents toward catechol are listed in Table 5. The results show that g-C_3_N_4_/Fe_3_O_4_ takes a comparatively shorter time to reach the equilibrium than other adsorbents such as activated carbon, modified dolomite, hydroxyapatite, α-alumina, magnetic vermiculite, resin, and waste Fe(III)/Cr(III) hydroxide. The quick adsorption on g-C_3_N_4_/Fe_3_O_4_ suggests that it has a very high adsorption efficiency of catechol from water in unit time. Furthermore, compared with other adsorbents, the presence of Fe_3_O_4_ on the g-C_3_N_4_/Fe_3_O_4_ composite makes the separation of catechol from water very rapidly by an external magnetic field, which greatly simplifies the cumbersome procedures such as centrifugation and filtration. Therefore, this reusable g-C_3_N_4_/Fe_3_O_4_ composite appears to be very effective and shows significant potential for removing catechol from water.

## 4. Conclusions

In this work, g-C_3_N_4_/Fe_3_O_4_ magnetic nanocomposite was successfully prepared by chemical co-precipitation and used as the adsorbent to separate catechol from water for the first time, presenting good adsorption properties such as considerably high capacity and short adsorption equilibrium time. More importantly, the g-C_3_N_4_−Fe_3_O_4_ nanocomposite could be recovered by an external magnetic field and reused without reducing the adsorption performance, even after five successive cycles. Therefore, g-C_3_N_4_−Fe_3_O_4_ nanocomposite is a promising material for the removal of catechol from water.

## Figures and Tables

**Table 1:** Experimental conditions during the adsorption of catechol.

Experimental Conditions	Concentration of Catechol (mg/L)	g-C_3_N_4_/Fe_3_O_4_ Dosage (mg)	Adsorption Time (min)	pH Value
Concentration of catechol (mg/L)	6, 10, 14, 20, 25, 30, 40, 50, 100, 200, 300, 400, 500, 600	50	30	6
g-C_3_N_4_/Fe_3_O_4_ dosage (mg)	10	12.5, 25, 37.5, 50, 75	30	7
Adsorption time (min)	10	50	5, 10, 20, 30, 40	7
pH value	10	50	30	4, 5, 6, 7, 8, 9

**Table 2:** Textural properties of g-C_3_N_4_ and the g-C_3_N_4_/Fe_3_O_4_ composite.

Materials	*S*_BET_ (m^2^/g)	Pore Volume (cm^3^/g)	Pore Size (nm)
g-C_3_N_4_	20.6	0.145	28.0
g-C_3_N_4_/Fe_3_O_4_	38.8	0.129	13.3

**Table 3:** Isotherm parameters for the adsorption of catechol on the g-C_3_N_4_/Fe_3_O_4_ composite.

Langmuir Isotherm Parameters		Freundlich Isotherm Parameters	
*Q*_max_ (mg/g)	24.9	*K*_F_ (L/mg)	0.101
*K*_L_ (L/mg)	0.00134	*n*	1.354
R^2^	0.9836	R^2^	0.9872

**Table 4:** Equations of the ionization of amine groups on the g-C_3_N_4_/Fe_3_O_4_ surface in an aqueous suspension [26,35,36].

pH of Solutions	Possible Ionization Equations
Acid conditions	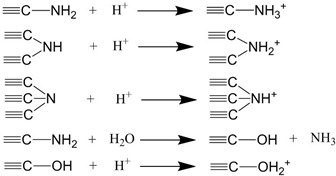
Base conditions	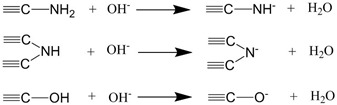

**Table 5:** Comparison of the adsorption performances of different adsorbents for catechol.

Adsorbent	*Q*_m_ (mg/g)	*t*_e_	*S*_BET_ (m^2^/g)	Ref.
Granular activated carbon	977.6	12 h	855	[12]
Activated carbon	238.10	48 h	1140	[13]
Modified dolomite	74.1	120 min	31.39	[14]
Modified montmorillonite	103.5796	30 min	-	[15]
Hydroxyapatite	15	120 min	145	[16]
Organophilic-bentonite	51.97	45 min	-	[17]
α-alumina	2.4	60 min	-	[21]
Magnetic vermiculite	75	240 min	37.5	[22]
Resin	160.75	150 min	-	[23]
Waste Fe(III)/Cr(III) hydroxide	4.0	100 min	156	[24]
g-C_3_N_4_/Fe_3_O_4_	24.9	30 min	38.8	This work
